# Circular DNA Intermediate in the Duplication of Nile Tilapia *vasa* Genes

**DOI:** 10.1371/journal.pone.0029477

**Published:** 2011-12-22

**Authors:** Koji Fujimura, Matthew A. Conte, Thomas D. Kocher

**Affiliations:** Department of Biology, University of Maryland, College Park, Maryland, United States of America; Auburn University, United States of America

## Abstract

*vasa* is a highly conserved RNA helicase involved in animal germ cell development. Among vertebrate species, it is typically present as a single copy per genome. Here we report the isolation and sequencing of BAC clones for Nile tilapia *vasa* genes. Contrary to a previous report that Nile tilapia have a single copy of the *vasa* gene, we find evidence for at least three *vasa* gene loci. The *vasa* gene locus was duplicated from the original site and integrated into two distant novel sites. For one of these insertions we find evidence that the duplication was mediated by a circular DNA intermediate. This mechanism of gene duplication may explain the origin of isolated gene duplicates during the evolution of fish genomes. These data provide a foundation for studying the role of multiple *vasa* genes in the development of tilapia gonads, and will contribute to investigations of the molecular mechanisms of sex determination and evolution in cichlid fishes.

## Introduction

Fish are the most species-rich group of vertebrates, making up more than half of the 55,000 vertebrate species [1]. Fish are an attractive group of organisms for studying the evolution of sex determination because members of this class exemplify a broad range of various types of sex determination and differentiation [2]. However, the molecular mechanisms by which sex is determined remain largely unclear.

The Nile tilapia *Oreochromis niloticus* is one of the most important cichlid fishes in aquaculture [3] and is also an excellent laboratory model for studies in physiology [4], [5], endocrinology [6], [7], genomic biology and molecular genetics [8]–[12] and developmental biology [13]–[15].

Cichlids in the East African Great Lakes are famous as spectacular examples of explosive adaptive radiation [16], [17]. The cichlids have various types of sex determination, and thus provide an opportunity to understand the molecular mechanisms of sex determination. Although there are no gross morphological differences in chromosome structure, sex is determined by a small number of genes in most species of cichlid examined to date [18], [19]. The patterns of gene expression in gonadal development of Nile tilapia have been extensively characterized [20].

We recently establish *Tol2*-mediated transgenesis in Nile tilapia with the ultimate goal of using germline-specific expressed promoter to reveal the molecular mechanism of sex differentiation *in vivo* [21]. One candidate for developing a gonad-specific promoter is the *vasa* (also called DDX4) gene. The *vasa* gene encodes a DEAD box (Asp-Glu-Ala-Asp) protein thought to be an ATP-dependent RNA helicase [22]. It was originally characterized in *Drosophila* [23], where it plays a critical role in specification of the germ cell lineage [24]. The regulatory regions of the teleost *vasa* gene have been used to control transgene expression in fish germ cells (zebrafish [25]; rainbow trout [26]; medaka [27]). The 3′-UTR of the Ostariophysan *vasa* mRNAs plays an important role in their localization to the germ cells [28].

Relatively little has been published about the *vasa* genes of the Nile tilapia. Kobayashi et al. [29] found that the expression pattern of the Nile tilapia *vasa* gene differed between male and female germ cells during gametogenesis. Kobayashi et al. [30] found two isoforms of *vasa* that were differentially expressed during the development of male and female gonads. They suggested that the isoforms were splicing variants of a single copy of the *vasa* gene.

In the current study, we isolated and sequenced BAC clones containing Nile tilapia *vasa* gene sequences. Contrary to the previous report, we find that Nile tilapia have at least three *vasa* gene loci, namely one original locus and two duplicated loci. We investigate their genomic structure, and discuss a duplication mechanism mediated by a circular DNA intermediate that appears to be responsible for at least one of the duplicated copies.

## Results

### BAC clones for Nile tilapia *vasa* gene

**Figure 1:**
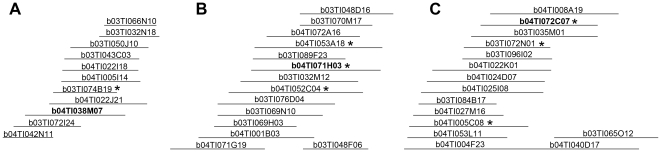
Three Nile tilapia BAC contigs contain *vasa* gene sequences. (A) Contig 311, (B) Contig 992 and (C) Contig 542. Asterisks indicate clones identified by a 4-step PCR screening for *vasa* gene sequences. Clones b04TI038M07 (38M07), b04TI071H03 (71H03), b04TI072C07 (72C07) were chosen for sequencing.

We used a 4-step PCR screening of pooled colonies to identify clones containing the *vasa* gene in two Nile tilapia BAC libraries [31], [32]. To determine the relationships among these clones, we next looked at contigs assembled from restriction fingerprint data of the BAC clones [32]. Surprisingly, we found that the candidate clones were subdivided into three fingerprint contigs (Figure 1). This result suggested that Nile tilapia have at least three distinct *vasa* gene loci. We chose three BAC clones, b04TI038M07 (hereafter written simply as 38M07), b04TI071H03 (71H03) and b04TI072C07 (72C07), from contigs 311, 992 and 542 respectively, for further sequence analysis.

### Full sequences of three BAC clones complement the Broad genomic data

We sequenced each of the three candidate clones on a Roche 454 DNA sequencer and assembled them using Newbler. The reads were assembled into 11 contigs for clone 38M07, 5 contigs for 71H03 and 3 contigs for 72C07. We then annotated the contigs by BLAST searching against the stickleback genomic data, and used this information to order the contigs. The final scaffold lengths of the BAC clone were 218,234 bp (38M07), 181,027 bp (71H03) and 189,421 bp (72C07) (Figure S1).

The Broad Institute recently released a first assembly of the Nile tilapia genome based on paired Illumina sequence reads (GenBank #PRJNA59571). We found the sequence of clone 38M07 matched Scaffold_160, 71H03 matched Scaffold_19, and 72C07 matched Scaffold_11 of this assembly (Figure S1). Our BAC data is largely consistent with the corresponding region of each genomic scaffold. However, the Broad genomic data partially lacks sequences for the *vasa* gene in each scaffold, while our BAC data contains all of the exons for *vasa* gene at each location (comparison to mRNA, Genbank Accession #AB032467, ref [29]; Figure S2). This implies that the Broad assembly of the genomic data did not accurately reconstruct the sequences of these recently duplicated genes.

### Nile Tilapia has one original and two extra loci for *vasa* gene

**Figure 2:**
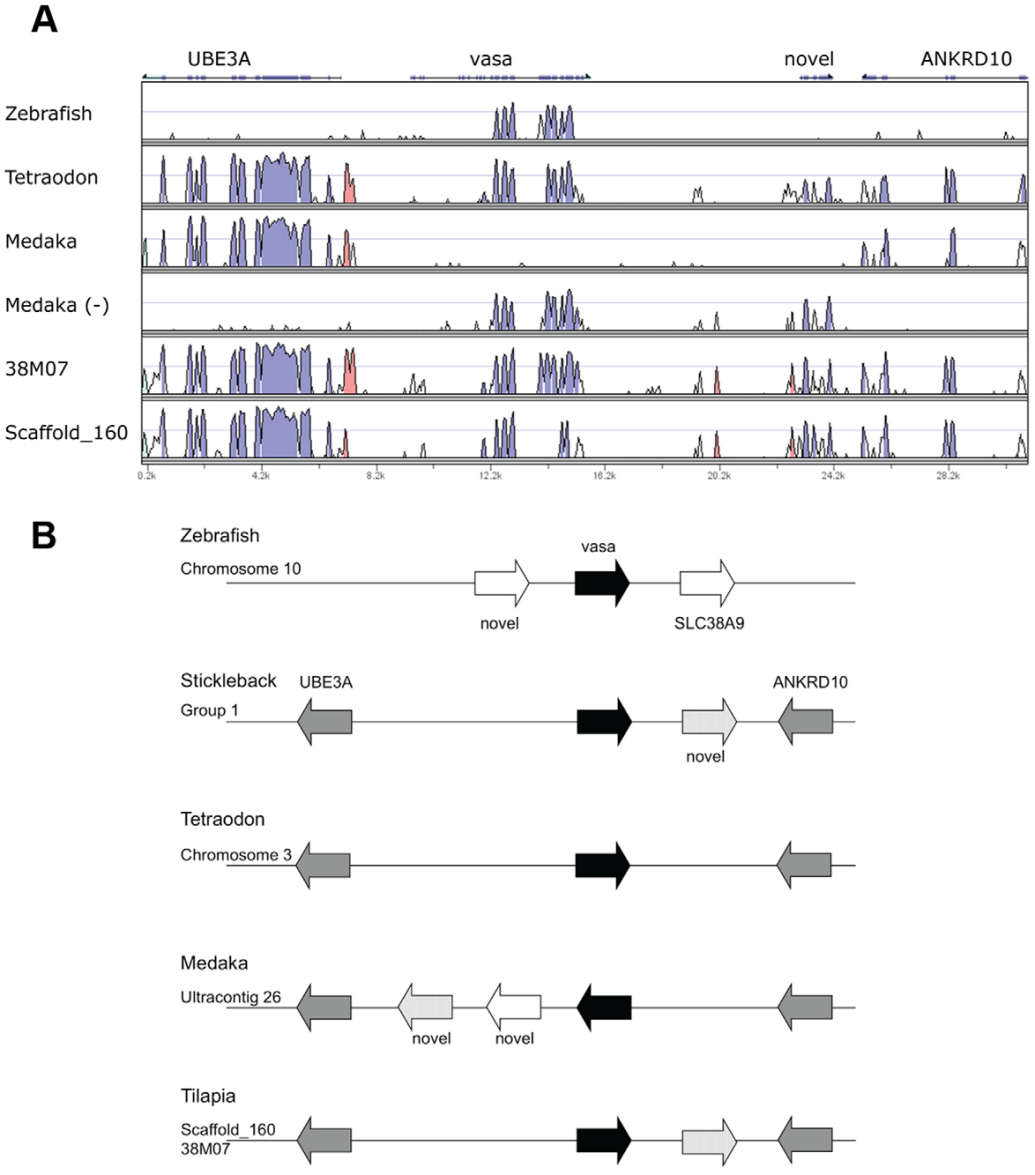
BAC clone 38M07 corresponds to a region between the UBE3A and ANKRD10 genes. (A) VISTA plots against stickleback show that 38M07 and Scaffold_160 cover a region between UBE3A and ANKRD10 genes, in which the *vasa* gene is located in other teleosts. The complementary sequence of medaka (-) was also used because of a local inversion of *vasa* in the genome assembly. (B) Genomic organization around the *vasa* gene of zebrafish, stickleback, *Tetraodon*, medaka and tilapia. The genomic organization is conserved in other teleosts except for the distantly related zebrafish, the inversion in medaka, and some lineage-specific predicted novel genes.

38M07 BAC clone and genome scaffold_160 have high similarity to a region between the ubiquitin protein ligase E3A (UBE3A) and ankyrin repeat domain 10 (ANKRD10) genes in other teleosts (Figure 2). Since the *vasa* gene of other teleosts is located in this region, clone 38M07 represents the original locus of the *vasa* gene. The genomic organization of this region is conserved among euteleosts including stickleback (*Gasterosteus aculeatus*), pufferfish (*Tetraodon nigroviridis*), and medaka (*Oryzias latipes*), except that the *vasa* gene is inverted in medaka, and that novel genes are predicted in some lineages. Stickleback, medaka and tilapia all have the same novel gene, a predicted protein with no similarity to other genes. The structure of this region is quite different in the more distantly related zebrafish (*Danio rerio*).

**Figure 3:**
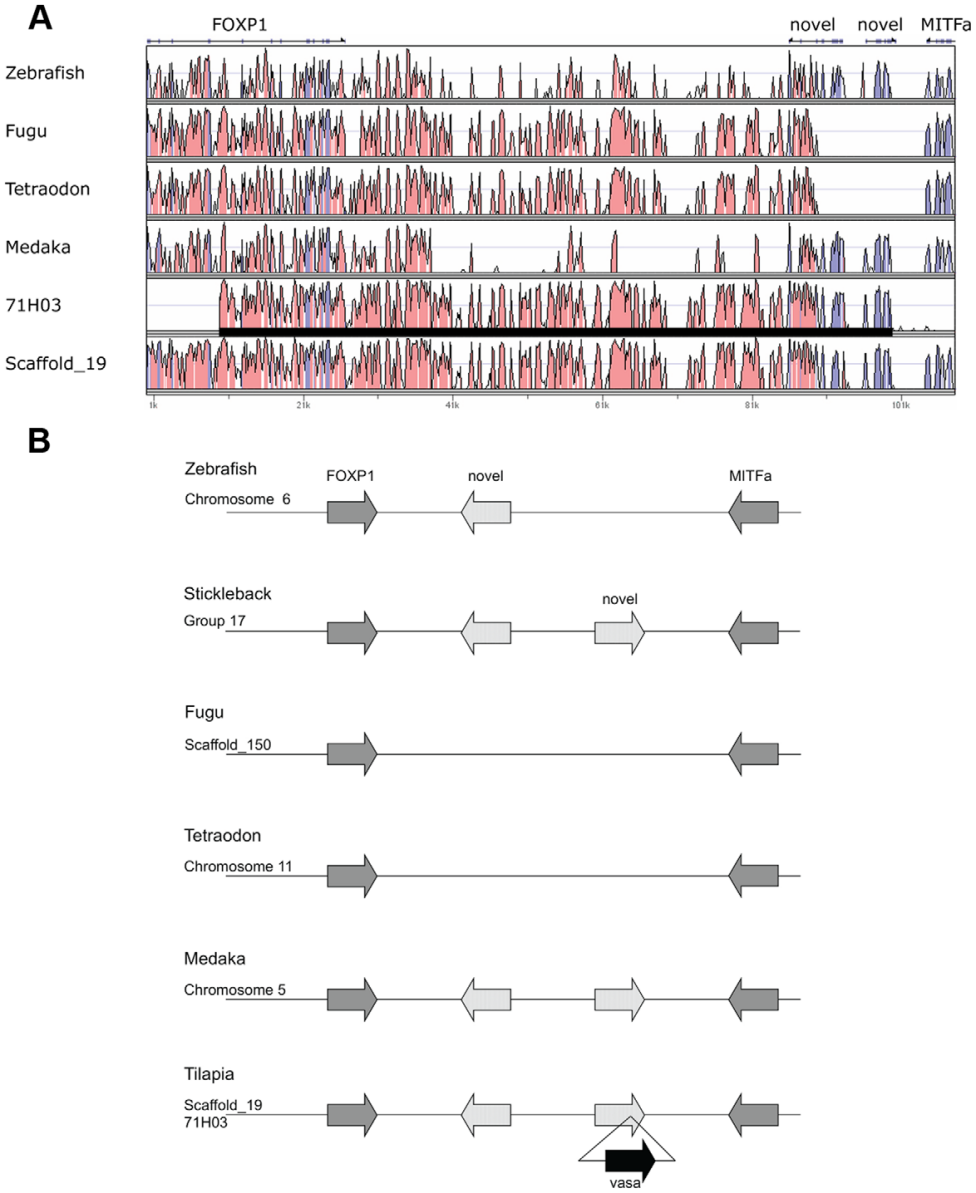
BAC clone 71H03 corresponds to a region between the FOXP1 and MITFa genes. (A) VISTA plots against stickleback show that 71H03 and Scaffold_19 correspond to a region between the FOXP1 and MITFa genes. Black bar indicates the region covered by BAC clone 71H03. Note that a *vasa* gene is not located in this region of other teleosts. (B) Genomic organization between the FOXP1 and MITFa genes of zebrafish, stickleback, fugu, *Tetraodon*, medaka and tilapia. The genomic organization is conserved in teleosts, except that two novel genes are predicted to be lineage-specific. The *vasa* gene copy on 71H03 was integrated into one of these novel genes.

BAC clone 71H03 and genome scaffold_19 are highly similar to a region between the forkhead box P1 (FOXP1) and the microphthalmia-associated transcription factor a (MITFa) genes in other teleosts (Figure 3). Since the *vasa* gene has not been observed in this location in any other teleosts, the sequence of 71H03 represents a duplication of the *vasa* gene. Aside from the insertion of the *vasa* gene, the genomic organization of this region is conserved among teleosts, except for two novel genes specific to particular lineages. The novel gene closest to FOXP1 is similar to the 3-hydroxybutyrate dehydrogenase type 2 (BDH2) gene, and has been lost in pufferfish (*Takifugu rubripes* and *Tetraodon nigroviridis*). The novel gene closest to MITFa is similar to the pentafunctional arom protein (ARO1) of yeast, and is found in stickleback, medaka and tilapia. The extra *vasa* gene was integrated into this second novel gene.

**Figure 4:**
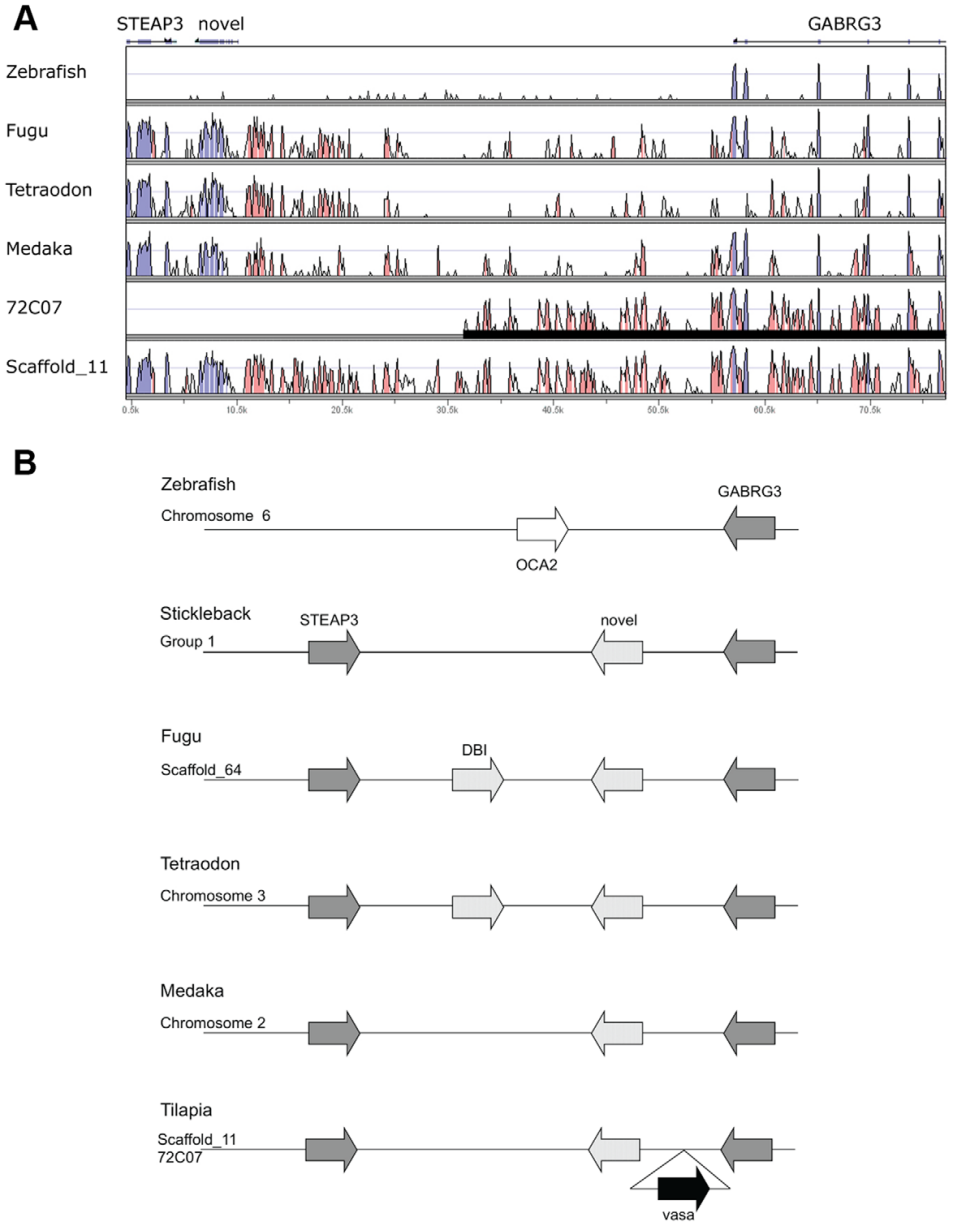
BAC clone 72C07 corresponds to a region between the STEAP3 and GABRG3 genes. (A) VISTA plots against stickleback show that 72C07 and Scaffold_11 correspond to a region between the STEAP3 and GABRG3 genes. Black bar indicates the region covered by BAC clone 72C07. Note that a *vasa* gene is not located in this region of other teleosts. (B) Genomic organization between the STEAP3 and GABRG3 genes of zebrafish, stickleback, fugu, *Tetraodon*, medaka and tilapia. The genomic organization is found conserved in other teleosts, except in the distantly related zebrafish and that the DBI gene is found to be lineage-specific. The *vasa* gene copy on 72C07 was inserted into the intergenic region between a novel gene and GABRG3.

BAC clone 72C07 and genome scaffold_11 have high similarities to a region between the six transmembrane epithelial antigen of the prostate (STEAP) family member 3 (STEAP3) and the gamma-aminobutyric acid (GABA) A receptor, gamma 3 (GABRG3) genes of other teleosts (Figure 4). As the *vasa* gene has not been observed in this region of other teleost genomes, this represents a second duplication of the *vasa* gene. The genomic organization is conserved among higher teleosts, except that the diazepam binding inhibitor (DBI) gene is present in *Takifugu* and *Tetraodon*. A novel gene similar to the secretin receptor was found in stickleback, *Takifugu*, *Tetraodon*, medaka and tilapia. The extra *vasa* gene was inserted into the intergenic region between this novel gene and GABRG3.

**Figure 5:**
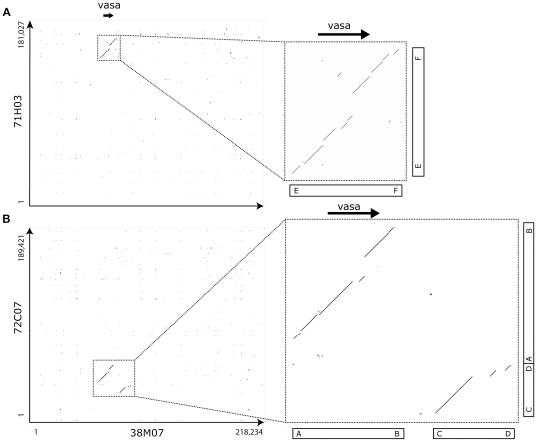
Only the *vasa* gene locus was duplicated in 71H03 and 72C07. (Left) Dotplots of 38M07 contigs against (A) 71H03 contigs and (B) 72C07 contigs. (Right) Magnification of the regions. Black arrow indicates the coding region of *vasa* gene. Boxes A–B and E–F show that only the *vasa* gene locus of 38M07 was duplicated into loci of 72C07 and 71H03, respectively. In addition, box C–D shows a difference in order in which the down stream region of the 38M07 *vasa* gene locus is duplicated into the upstream region of the 72C07 *vasa* gene locus. The boxes A–B, C–D and E–F correspond to those of Figures 7, 8, S3, S4 and S5.

We then compared the sequences of clone 38M07 to 71H03 and 72C07 (Figure 5). Dotplots show that only a small region around the original *vasa* locus was inserted into 71H03 (Box E–F of Figure 5A) and 72C07 (Box A–B of Figure 5B). The dotplot further shows a difference in the order of segments in 72C07. The downstream region of the 38M07 *vasa* locus is duplicated into the upstream region of the 72C07 *vasa* locus (Box C–D of Figure 5B). This means that the three loci each have an intact gene, and that the regions around them are different.

We predicted the exon-intron structure of *vasa* gene in all three BAC clones by comparison to the putative full-length tilapia mRNA sequence, which is deposited in Genbank (Accession #AB032467, ref [29]; Tables S1, S2, S3). We predicted that the three BAC clones possess 22 exons for the coding region of *vasa* gene, and that the 5′-UTR of the mRNA sequence is on two exons (exon1, and part of exon 2). For 38M07 and 72C07, the 3′-UTR of the cDNA sequence followed after the stop codon on a part of exon 23 and continued on exon 24, while in 71H03 exons 23 and 24 are adjacent without an intron. The predicted *vasa* gene sequences of the three BAC clones were slightly different from that of the mRNA (38M07 98.0%; 71H03 98.9%; 72C07 97.1%). Differences were also found in the lengths of exons 6, 10, 12 and 13 (Tables S1, S2, S3). While the exons in clones 38M07 and 72C07 are in frame, the short exon 6 of 71H03 generates a stop codon in exon 7. Therefore, the *vasa* gene on clone 71H03 might be a pseudogene, or might produce the short splicing form, which skips exons 6 and 8 (see *Discussion*).

## Discussion

**Figure 6:**
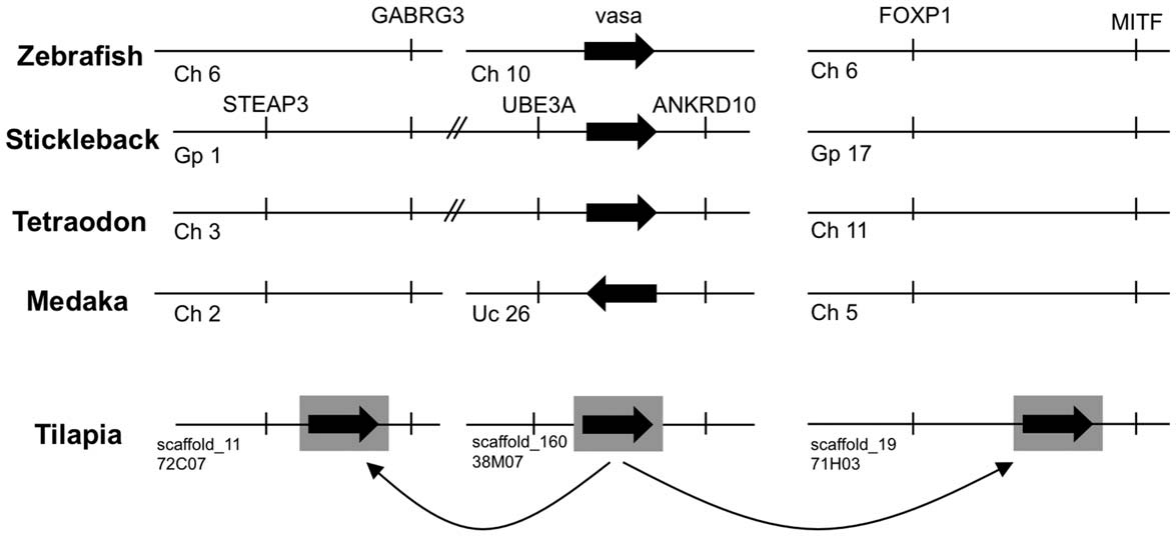
Nile tilapia has one ancestral and two recent duplicate *vasa* loci. This study reveals that Nile tilapia has at least three *vasa* loci with one original and two extra loci for *vasa* gene. In these duplications, only a small region encompassing the *vasa* gene was duplicated from the original site and integrated into novel sites.

Taken together, these results suggest that the Nile tilapia have at least three copies of the *vasa* gene. The locus at the ancestral site has been duplicated to create two additional *vasa* genes located at distant sites (Figure 6). Importantly, the duplicated loci retain the introns of the original locus, excluding the possibility that these duplicates were created by reverse transcription of a *vasa* mRNA. The duplicated loci also retain upstream regions of several kilobases from the putative transcriptional start sites of exon 1. Therefore they are likely to be functional for the regulation of gene expression. While the copy on BAC 72C07 was inserted into an intergenic region, the copy on BAC 71H03 was inserted into an existing novel gene locus. It is not known whether the insertion in 71H03 knocked out the existing gene function or produced a new hybrid gene product. Further studies are needed to determine whether each *vasa* locus produces a functional gene and how these insertions have affected the expression of neighboring genes.

Kobayashi et al. [30] reported that Nile tilapia have two isoforms of the *vasa* gene. The short form (Vas-s) was identical with the normal one (Vas) except that it lacked two small portions of the N-terminal regions, which were predicted as exons 6 and 8 in this study (Table S4). We found that the exons on clones 38M07 and 72C07 are in frame throughout the coding region. Therefore, *vasa* genes on clones 38M07 and 72C07 could be functional. On the other hand, the short exon 6 of clone 71H03 generates a stop codon in exon 7. However, if the exons 6 and 8 of clone 71H03 were skipped like Vas-s, then the short exons could be in frame. Therefore, we could not determine whether 71H03 is a pseudogene, or produces the short splicing form. Further studies are needed to determine how the splicing isoforms are generated.

Most chordates have a single copy of the *vasa* gene, although alternative splicing forms have been characterized in some species (e.g. zebrafish [33]). A dominant feature of fish genomes is the whole genome duplication that occurred at the origin of teleost fish [34], followed by lineage-specific gains and losses of individual genes [35]. Sequence polymorphisms in *vasa* likely arising from gene duplicates have been characterized in goldfish *Carassius auratus* (Accession Numbers: AY821683, AY821684) and swamp eel *Monopterus albus* (AY912131, DQ174775). Therefore, it is likely that lineage-specific duplication of *vasa* gene has occurred several times during the evolution of teleosts. Draft genome assemblies of four East African cichlids closely related to tilapia (i.e. *Astatotilapia burtoni*, *Metriaclima zebra*, *Pundamilia nyererei* and *Neolamprologus brichardi*) have only one copy of the *vasa* gene (Figure S3). The divergence time between tilapia and the East African cichlids is estimated at 10 million year [17]. The synonymous substitution rate of 38M07-71H03 and 38M07-72C07 were calculated at 0.0152 and 0.0170, respectively. If the synonymous substitution rate per year is assumed to be 5.5×10^−9^ [36], the time of divergence between 38M07-71H03 and between 38M07-72C07 would be estimated at 2.76 and 3.09 million year, respectively. Therefore, the duplications of the *vasa* gene we have characterized in Nile tilapia might have occurred twice during the recent evolution of the genus *Oreochromis*: the duplication of 72C07 was first, and 71H03 second.

We found that Nile tilapia has undergone duplication of the *vasa* gene by an unusual mechanism, in which a large fragment encompassing the coding region was duplicated from the original site and integrated in novel sites. Retention of the ancestral exon-intron structure in the duplicated loci indicates the duplication was via a DNA intermediate, not by reverse transcription of an mRNA. The structure of the insertion in 72C07 suggests a circular intermediate in the duplication. Circular DNA intermediates have been recognized recently as a new mechanism to explain eukaryotic gene duplication. Borneman et al. [37] characterized the genome of industrial strains of yeast *Saccharomyces cerevisiae*, and found a cluster of five ORFs have integrated into the genomes at multiple points via circular DNA intermediates, whose length is estimated to be around 15 kb. Durkin et al. [38] also found a segment of the KIT gene that is involved in coloring animal coats, was duplicated via circular DNA intermediates, whose length is estimated to be less than 480 kb, and concluded that it would cause coat color changes in some breeds of cattle.

**Figure 7:**
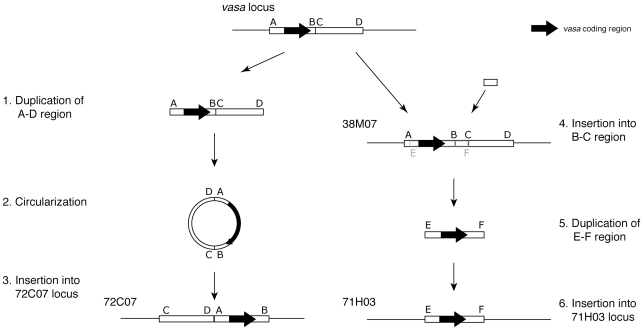
A model for the duplication of a Nile tilapia *vasa* gene. Duplications of the *vasa* gene occurred twice during the recent evolution of the genus *Oreochromis*. We speculated that the B–C region was inserted into 38M07 shortly after the duplication of 72C07. A circular DNA intermediate, which is recently recognized as a new mechanism to explain gene duplication, can explain the generation of a novel arrangement on clone 72C07. On the other hand, we have no evidence that the *vasa* gene in clone 71H03 was duplicated via a circular intermediate. Black arrow indicates the coding region of *vasa* gene. Boxes A–B, C–D, and E–F correspond to those of Figures 5, 8, S3, S4 and S5.

The difference in sequence order between clones 38M07 and 72C07 can be explained by this new mechanism. We believe that the duplication of at least the 72C07 *vasa* gene occurred via a circular DNA intermediate, whose length was about 28 kb (Figure 7). Since B–C region was not found in the *vasa* gene loci of the East African cichlids (dot-lined box in Figure S3), we speculated that the B–C region was inserted into 38M07 sometime between the duplication of 72C07 and 71H03. Several copies of the 6 bp motif GCAAAC were found around B, and might be involved in the insertion of B–C region (Figure S4).

**Figure 8:**
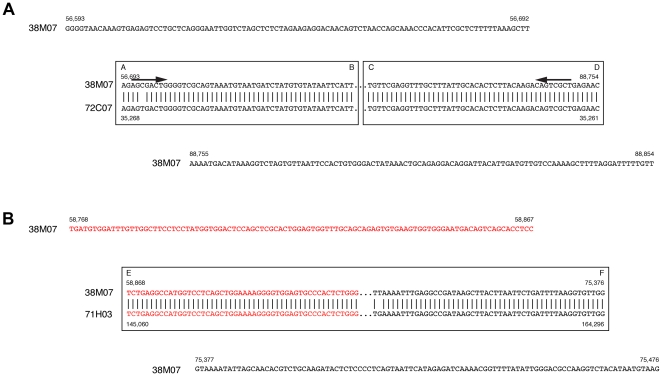
Sequences of the duplication boundaries. (A) 100 bp of 38M07 sequences flanking the left side of Box A–B and the right side of Box C–D. Black arrows indicate an 8 bp inverted repeat at sites A and D. (B) 100 bp of 38M07 sequences flanking Box E–F. Red letters indicate an *Expander* LINE, shown in Figure S5. Boxes A–B, C–D, and E–F correspond to those of Figures 5, 7, S3, S4 and S5.

Eichler et al. [39] found the motif CAGGG near the breakpoints in duplicated human loci, and speculated that the motif would be evidence for duplication model by circular DNA intermediate. We could not find any similar motifs in the sequences of the duplication boundaries (Figure 8 and Figure S4). However, an 8 bp inverted repeat was found in sites A and D of 38M07 (Figure 8A). We speculate that this 8 bp sequence was involved in generating circular DNA intermediates during the duplication.

We do not know whether the *vasa* gene in clone 71H03 was duplicated via a circular intermediate, because the signature inversion of sequence order was not found. We found a fragment of *Expander* (also known as *Rex3* [40]) at the starting sites of 38M07 and 71H03 (red in Figures 8B, also Figure S5), and it could be speculated that this LINE mediated the duplication of 71H03 *vasa* gene locus.

Our study has discovered evidence for two different mechanisms that might play an important role in lineage specific gene duplication in fishes. Further, we have characterized the regulatory regions flanking all three copies of the *vasa* gene in the Nile tilapia genome, which will allow the design of transgenic constructs for specific expression of genes in the developing gonad.

## Materials and Methods

### Screening, sequencing, and analysis of Nile tilapia BAC clones

To determine the genomic sequence of the Nile tilapia *vasa* genes, we screened a BAC library derived from sperm of the Lake Manzallah strain of *O. niloticus* [31]. We performed a PCR-based BAC library screening [32], using primers vasaF (5′- GGC AAA TGT TCT GTC CTG GT -3′) and vasaR (5′- CAC TGT CAG CTC CTG GAT CA -3′), designed using tilapia cDNA sequence data and targeting an exon that is highly conserved among teleosts. Overlapping BACs were identified from the contigs assembled from restriction fingerprint data [32]. Selected BAC clones were cultured and prepared using the PSI Clone Big BAC DNA kit (Princeton Separations, Freehold NJ, USA). Sequences of these clones were determined on a Roche 454 DNA sequencer (Branford CT, USA) and assembled using Newbler [41]. GenBank accession numbers of the assembled clones are AB649031-3.

Blast searches against the recently released tilapia genome assembly (GenBank accessiong #PRJNA59571) were done using the bioinformatic resources of BouillaBase.org (http://cichlid.umd.edu/blast/blast.html). Alignments of the BAC clones to the genomic sequences were constructed with PipMaker [42].

### Genomic structure for three loci of *vasa* gene

The genomic information for teleost vasa regions was downloaded from Ensembl (http://www.ensembl.org). To construct Figure 2, we analyzed the region 6,543,795–6,575,246 bp on chromosome 10 of zebrafish (Zv9), the region 26,979,595–27,010,559 bp on group 1 of stickleback (BROADS1), the region 7,504,473–7,530,546 bp on chromosome 3 of *Tetraodon*, (TETRAODON8), and the region 15,196–58,492 bp on ultracontig 26 of medaka, (MEDAKA1). The genomic data of *Takifugu* (FUGU4) has not been assembled between ENSTRUG00000006601 on scaffold_284 and ENSTRUG00000002557 on scaffold_151, so it was not included in our analysis. For Figure 3, we analyzed the region 47,919,490–47,463,463 bp on chromosome 6 of zebrafish, 13,964,270–14,072,374 bp on stickleback group 17; 516,574–618,053 bp on scaffold_150 of fugu; 10,990,779–11,069,617 bp on chromosome 11 of *Tetraodon* and 4,992,801–5,209,014 bp on chromosome 5 of medaka. For Figure 4, we compared the region 37,813,999–38,288,577 bp on zebrafish chromosome 6, 23,562,074–23,639,703 bp on stickleback group 1, 835,659–940,881 bp on *Takifugu* scaffold_64, 4,433,359–4,514,716 bp on *Tetraodon* chromosome 3 and 24,776,513–24,977,787 bp on medaka chromosome 2. We used VISTA ([43], AVID alignment method, unmasked sequences) to align and visualize the genomic sequences. For Figure S5, repetitive elements of tilapia genome were identified with RepeatModeler (http://www.repeatmasker.org/RepeatModeler.html).

## Supporting Information

Figure S1 **Comparison of BAC sequence contigs with the Broad genome assembly.** Dotplots of the BAC sequence contigs against equivalent genomic scaffolds. (A) Orientation of 11 contigs for 38M07 against genomic scaffold_160. (B) 5 contigs of 71H03 against scaffold_19. (C) 3 contigs of 72C07 against scaffold_11. Note that regions containing *vasa* gene sequences (arrow) are poorly matched.
(TIF)

Figure S2 **Comparison of** ***vasa*** **mRNA sequences to the BACs and genome scaffolds.** (A) Dotplots of the vasa mRNA sequence (Genbank accession #AB032467 [29]) versus BAC 38M07 and genome scaffold_160. (B) *vasa* mRNA versus BAC 71H03 and genome scaffold_19. (C) vasa mRNA versus BAC 72C07 and genome scaffold_11. Note that all of the exons can be predicted in the comparisons of the mRNA to the BAC sequences, but that not all of the exons are predicted from the genome scaffolds.
(TIF)

Figure S3 **Comparison of Nile tilapia 38M07 with draft genome assemblies of four East African cichlids.** (A) *Neolamprologus brichardi*, (B) *Astatotilapia burtoni*, (C) *Metriaclima zebra*, (D) *Pundamilia nyererei*. Black arrow indicates the coding region of *vasa* gene. Boxes A–B and C–D correspond to those of Figures 5, 7, 8, S4 and S5. Dot-lined box indicates that the four cichlids are missing the B–C region and part of the C–D region. Black arrow indicates the coding region of *vasa* gene.
(TIF)

Figure S4 **Sequences of the duplication boundaries.** (A) 100 bp of 71H03 sequence flanking Box E–F. Red and green letters indicate a LINE of *Expander* and a DNA transposon of Tc1-like respectively, as shown in Figure S5. (B) 100 bp of 72C07 sequences flanking the left side of Box C–D and the right side of Box A–B. (C) 100 bp of 38M07 and 71H03 sequences flanking Box B–C. Blue arrows indicate a 6 bp motif of GCAAAC. Boxes A–B, C–D and E–F correspond to those of Figures 5, 7, 8, S3 and S5.
(TIF)

Figure S5 **Repetitive elements around** ***vasa*** **gene loci.** Fragments of repetitive elements were shown as colored boxes for (A) 38M07, (B) 71H03 and (C) 72C07. Black arrow indicates the coding region of *vasa* gene. Boxes A–B, C–D and E–F correspond to those of Figures 5, 7, 8, S3 and S4. Fragments of the long interspersed element (LINE) *Expander* (also known as *Rex3*) were found at the breakpoints in 38M07 and 71H03.
(TIF)

Table S1 **Predicted exons of 38M07** ***vasa*** **gene.**
(XLS)

Table S2 **Predicted exons of 71H03** ***vasa*** **gene.**
(XLS)

Table S3 **Predicted exons of 72C07** ***vasa*** **gene.**
(XLS)

Table S4 **Predicted exons of mRNA sequences, which are deposited on GenBank.**
(XLS)
