# Supplementary figures and images for: Circular DNA Intermediate in the Duplication of Nile Tilapia vasa Genes
Source: PLoS One. 2011 Dec 22;6(12):e29477. doi: 10.1371/journal.pone.0029477 (PMC3245284; doi:10.1371/journal.pone.0029477)

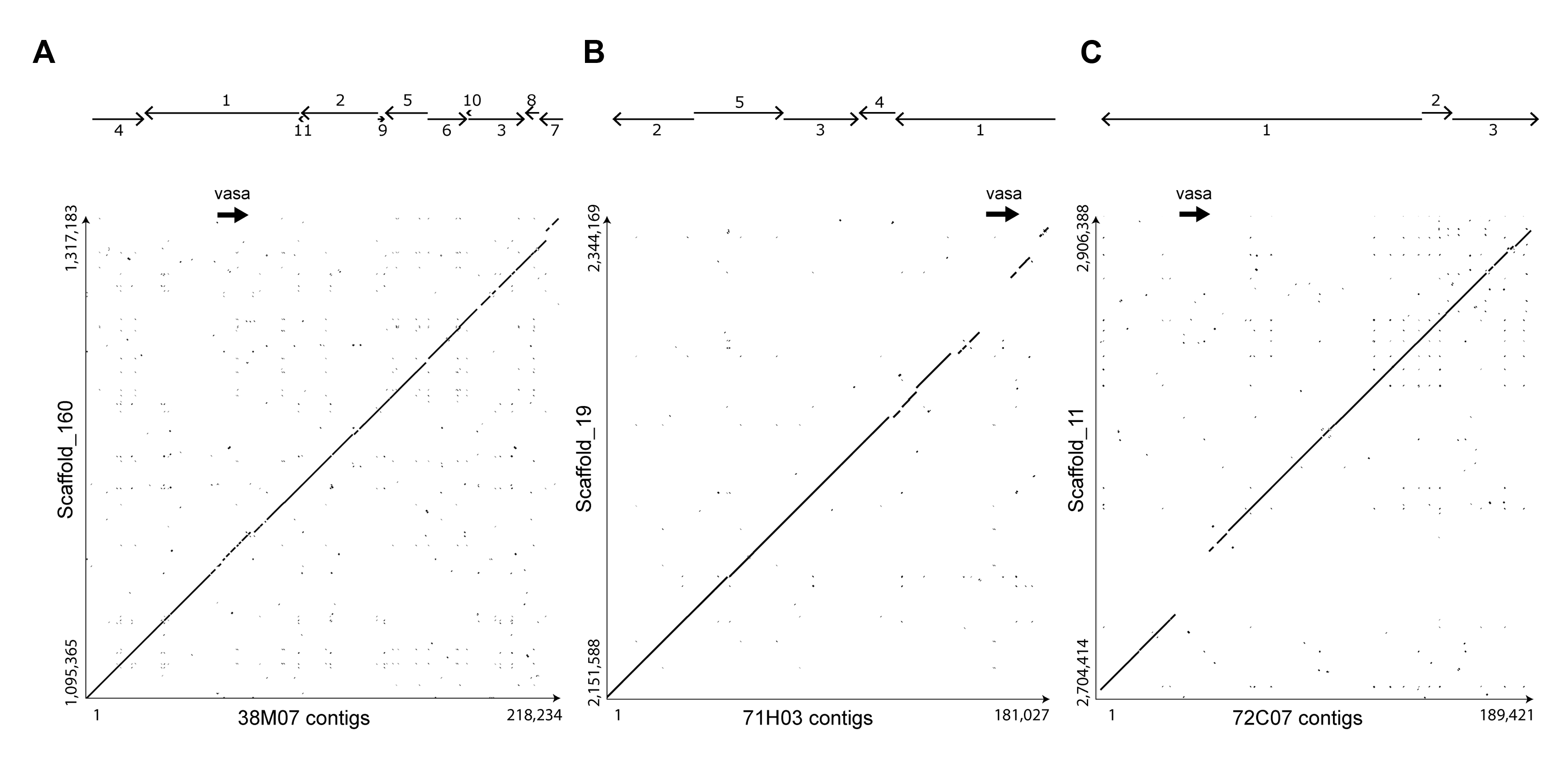

Supplement: Figure S1 — Comparison of BAC sequence contigs with the Broad genome assembly. Dotplots of the BAC sequence contigs against equivalent genomic scaffolds. (A) Orientation of 11 contigs for 38M07 against genomic scaffold_160. (B) 5 contigs of 71H03 against scaffold_19. (C) 3 contigs of 72C07 against scaffold_11. Note that regions containing vasa gene sequences (arrow) are poorly matched. (TIF) [file pone.0029477.s001.tif]

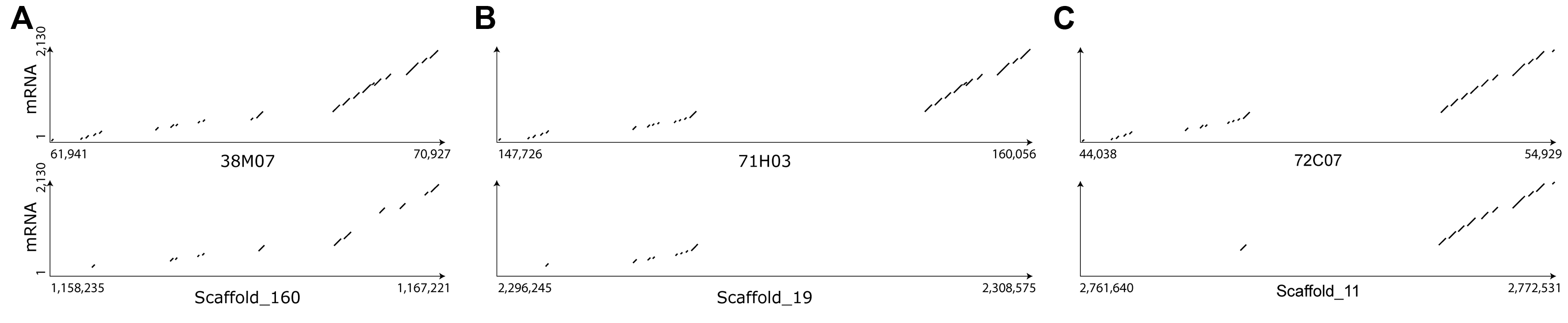

Supplement: Figure S2 — Comparison of vasa mRNA sequences to the BACs and genome scaffolds. (A) Dotplots of the vasa mRNA sequence (Genbank accession #AB032467 [29]) versus BAC 38M07 and genome scaffold_160. (B) vasa mRNA versus BAC 71H03 and genome scaffold_19. (C) vasa mRNA versus BAC 72C07 and genome scaffold_11. Note that all of the exons can be predicted in the comparisons of the mRNA to the BAC sequences, but that not all of the exons are predicted from the genome scaffolds. (TIF) [file pone.0029477.s002.tif]

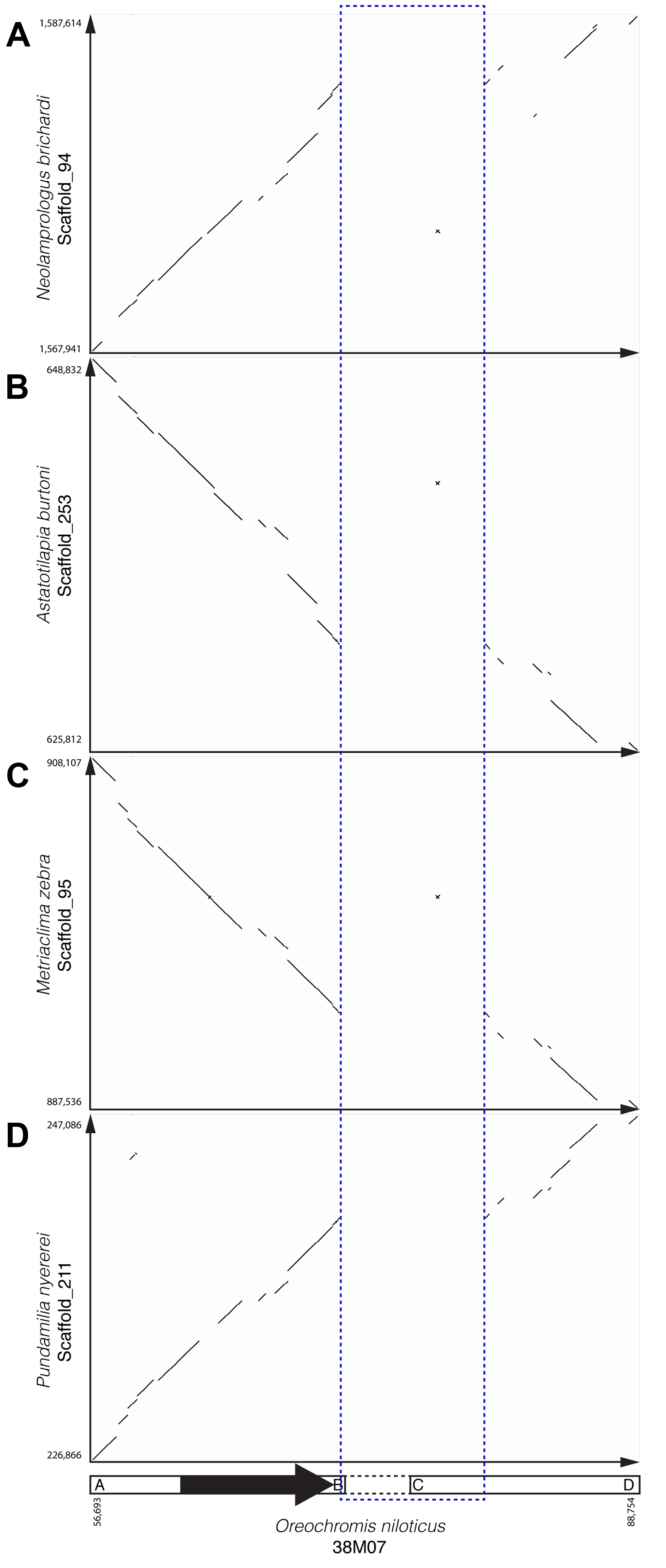

Supplement: Figure S3 — Comparison of Nile tilapia 38M07 with draft genome assemblies of four East African cichlids. (A) Neolamprologus brichardi, (B) Astatotilapia burtoni, (C) Metriaclima zebra, (D) Pundamilia nyererei. Black arrow indicates the coding region of vasa gene. Boxes A–B and C–D correspond to those of Figures 5, 7, 8, S4 and S5. Dot-lined box indicates that the four cichlids are missing the B–C region and part of the C–D region. Black arrow indicates the coding region of vasa gene. (TIF) [file pone.0029477.s003.tif]

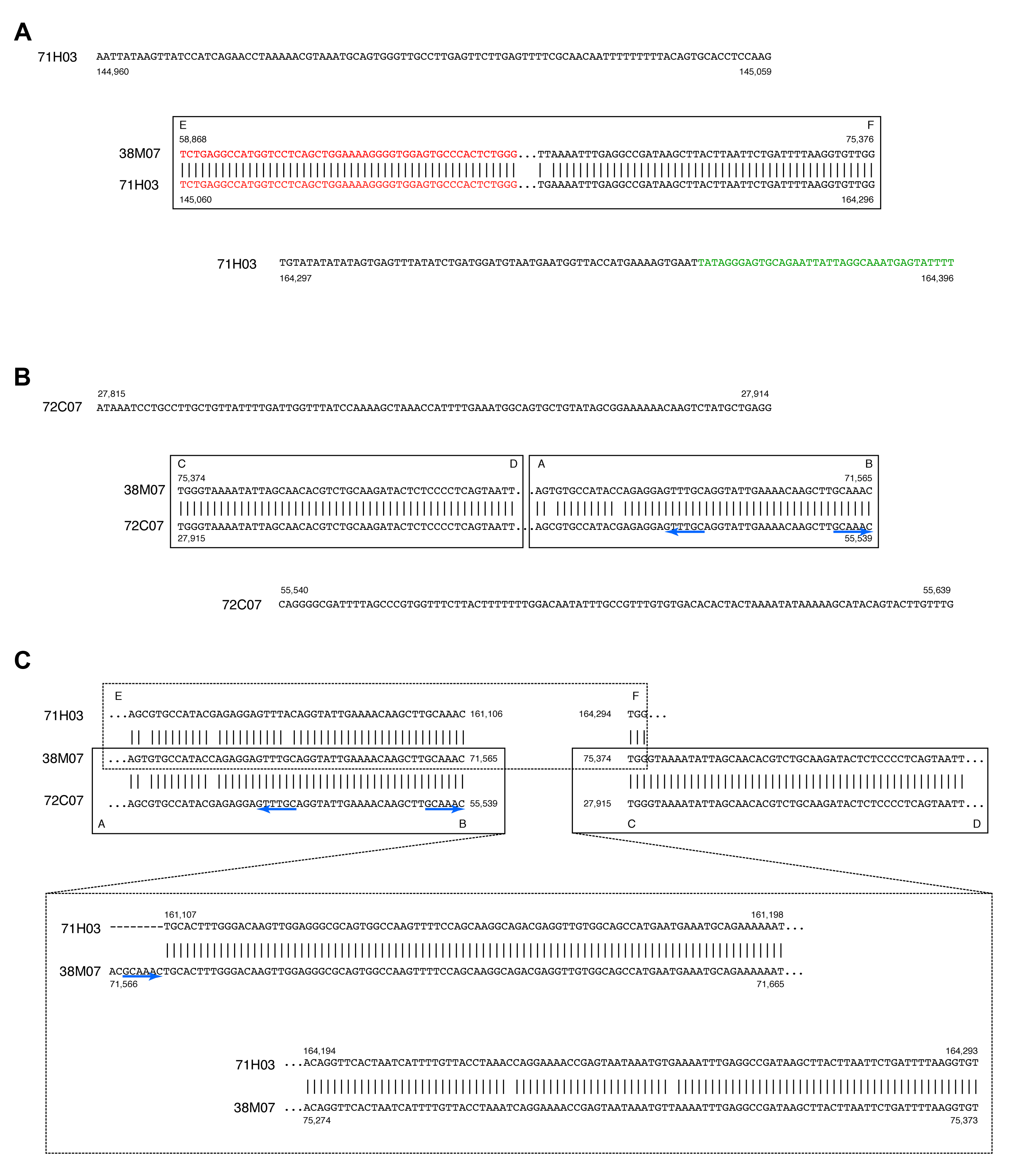

Supplement: Figure S4 — Sequences of the duplication boundaries. (A) 100 bp of 71H03 sequence flanking Box E–F. Red and green letters indicate a LINE of Expander and a DNA transposon of Tc1-like respectively, as shown in Figure S5. (B) 100 bp of 72C07 sequences flanking the left side of Box C–D and the right side of Box A–B. (C) 100 bp of 38M07 and 71H03 sequences flanking Box B–C. Blue arrows indicate a 6 bp motif of GCAAAC. Boxes A–B, C–D and E–F correspond to those of Figures 5, 7, 8, S3 and S5. (TIF) [file pone.0029477.s004.tif]

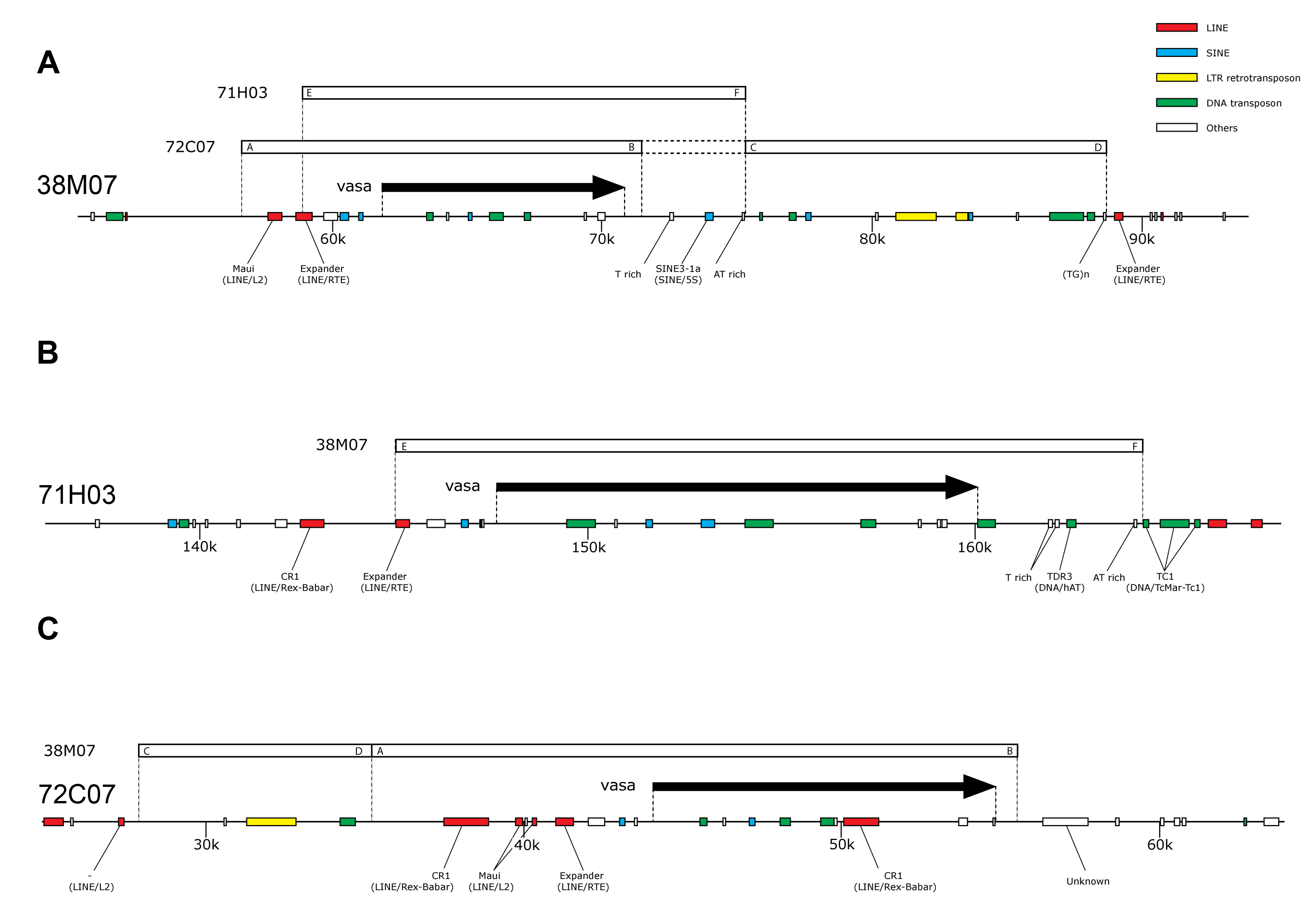

Supplement: Figure S5 — Repetitive elements around vasa gene loci. Fragments of repetitive elements were shown as colored boxes for (A) 38M07, (B) 71H03 and (C) 72C07. Black arrow indicates the coding region of vasa gene. Boxes A–B, C–D and E–F correspond to those of Figures 5, 7, 8, S3 and S4. Fragments of the long interspersed element (LINE) Expander (also known as Rex3) were found at the breakpoints in 38M07 and 71H03. (TIF) [file pone.0029477.s005.tif]
